# Bioreactor Production Process of *Spodoptera frugiperda multiple nucleopolyhedrovirus* Biopesticide

**DOI:** 10.3390/pathogens12081001

**Published:** 2023-07-31

**Authors:** Karina Klafke, Marcio Martinello Sanches, William Sihler, Marlinda Lobo de Souza, Aldo Tonso

**Affiliations:** 1 Department of Chemical Engineering, Polytechnic School, University of Sao Paulo, Sao Paulo 05508-010, SP, Brazil; kk.klafke@outlook.com; 2 Embrapa Beef Cattle, Campo Grande 79106-550, MS, Brazil; marcio.sanches@embrapa.br; 3 Embrapa Recursos Genéticos e Biotecnologia, Brasilia 70770-917, DF, Brazil; william.sihler@embrapa.br (W.S.); marlinda.souza@embrapa.br (M.L.d.S.)

**Keywords:** SfMNPV, *Spodoptera frugiperda*, in vitro production, fall armyworm, baculovirus, biological control, bioreactor

## Abstract

*Spodoptera frugiperda* (fall armyworm) is one of the most important maize pests in the world and the baculovirus *Spodoptera frugiperda multiple nucleopolyhedrovirus* (SfMNPV), a natural pathogen of this pest, has been used as a biopesticide for its control. At present, in vivo strategies at the commercial scale are employed by multiplying the virus in the host insect in biofactory facilities; however, in vitro large-scale production is an interesting alternative to overcome the limitations of baculoviruses massal production. This study aimed to develop the process of the SfMNPV in vitro production by evaluating the effects of different multiplicities of infection (MOI) and nutritional supplements, morphological and molecular analysis of the infection on the growth of Sf9 cells and virus production. The Bioreactor Stirred Tank Reactor (STR) approach with glutamine-supplemented Sf-900 III serum free culture medium, combined with the MOI of 1.0, showed the best viral production performance, with a specific productivity above 300 occlusion bodies (OBs)/cell and volumetric productivity of 9.0 × 10^11^ OBs/L.

## 1. Introduction

The fall armyworm (FAW) *Spodoptera frugiperda* (J. E. Smith) (Lepidoptera: Noctuidae) is native to the tropical regions of the Western Hemisphere, from the United States to Argentina [1]. It is a cosmopolitan polyphagous noctuid and considered the most important insect pest in maize crops worldwide, with recently reported occurrences in Africa and Asia continents, causing significant damage at all stages of plant development [2,3,4]. This pest has been mainly controlled with chemical pesticides or genetically modified maize, raising environmental concerns and generating resistant biotypes [1,5,6]. In this context, baculoviruses have been considered as an alternative for the control of FAW. The *Spodoptera frugiperda multiple nucleopolyhedrovirus* (SfMNPV), genus *Alphabaculovirus*, family *Baculoviridae*, is pathogenic to FAW. Some isolates of *Spodoptera frugiperda multiple nucleopolyhedrovirus* (SfMNPV), genus *Alphabaculovirus*, family *Baculoviridae*, have been successfully used as biopesticides for the control of this pest in many countries [7,8,9,10]. The production of SfMNPV for use as a commercial biopesticide is obtained by infecting healthy larvae but it has some disadvantages [11,12]. However, baculovirus multiplication in cell culture has the potential to overcome these limitations during large-scale production [13]. The SF-21 and Sf9 cell lines support the wild SfMNPV isolates’ replication both in suspension and in static culture [14,15]. One of the downsides of the serial passage of virus in insect cell culture is the occurrence of Few-Polyhedra (FP) mutants that produce low yields of occlusion bodies (OBs), which have an advantageous rate of virus replication [16] but show a low virulence of SfMNPV produced in SF-21 cells [17]. At the laboratory scale, the in vitro production of SfMNPV is considered commercially viable when it reaches 300 Occlusion Bodies (OBs)/cell [13]. Therefore, this work aimed to scale up the in vitro production of SfMNPV, optimizing cell growth and viral inoculum parameters and comparing the OBs production with different MOIs to increase the final benefit–cost ration.

## 2. Materials and Methods

### 2.1. Cell Line, Virus, and Medium

*Spodoptera frugiperda* Sf9 cell line (ATCC code 12659-017) stock was maintained at 28 °C in Sf-900 III insect cell culture medium (Life Technologies, Carlsbad, CA, USA) in 0.5 L Schott bottles under 130 rpm agitation speed in an orbital shaker (Innova 4000, New Brunswick Scientific, Edison, NJ, USA). The virus isolate used in this study, SfMNPV-19, was obtained from *S. frugiperda* [18] and was kindly provided by Dr. Fernando Hercos Valicente (Embrapa Milho e Sorgo). This isolate is deposited in the Embrapa Invertebrate Virus Collection (CVI), in Brasilia, Brazil. (CVI code BRM5023). The virus stock suspension of 4.65 × 10^6^ pfu/mL was previously obtained from static culture [15]. All cell cultures used Sf-900™ III Serum Free Medium (Gibco, Waltham, MA, USA).

### 2.2. Evaluation of Culture Media Supplementation for Sf9 Cells Growth

The cells were grown in a Bioreactor Stirred Tank Reactor (STR) BioFlo 110 (New Brunswick Scientific) with a working volume of 1.0 L. The following parameters were adopted: temperature 28 °C, Dissolved Oxygen (DO) 30% of air saturation, agitation 80 rpm, aeration flowrate 200 mL/min, and initial cell concentration 5 × 10^5^ cells/mL. A batch without supplementation (Run 1) and a batch with glutamine supplementation (Run 2) were used. In the supplemented batch, glutamine (Merck) was added to the culture medium at a proportion of 1.0 g/L at the end of the exponential growth phase. Cell cultures were monitored for growth and metabolic activity parameters.

### 2.3. Effect of Multiplicity of Infection (MOI) on SfMNPV In Vitro Production

The influence of two different multiplicities of infection (MOIs) in the SfMNPV production was investigated in bioreactor BioFlo 110 (New Brunswick Scientific) or Biostat B (Sartorius, Göttingen, Germany), with the previously described parameters. The culture medium was supplemented with glutamine 1.0 g/L at the beginning of cell growth. The viral inoculum was tittered by the serial dilution method [19] and the medium tissue culture infectious dose (TCID_50_) value was calculated according to [20]. The virus infection was carried out when the cells reached the concentration of 3.5 × 10^6^ cells/mL. One batch was implemented with an MOI of 0.1 (Run 3), and another batch with an MOI of 1.0 (Run 4). Cell growth was monitored for metabolic activity and viral infection parameters for 11 days in the batch with MOI 0.1 and 17 days in the batch with MOI 1.0.

### 2.4. Analysis of Growth, Nutrients and Metabolites

Each bioreactor batch typically collected samples twice a day for cells, glucose, glutamine, glutamate, lactate, and ammonium quantification. The cells were counted in a Neubauer chamber under phase-contrast microscopy at 200×. The viable cells were quantified with 0.4% Trypan blue (Sigma-Aldrich, San Luis, MO, USA). The samples were centrifuged at 3000× *g* for 3 min. The supernatant was collected and processed in the biochemistry analyzer YSI 2700 Select (Yellow Springs Instruments) according to the manufacturer’s instructions, except for ammonium quantification. In this case, the supernatant was quantified by colorimetric kit Genese (BioAssay Systems, Hayward, CA, USA) according to the manufacturer’s instructions, with readings carried out in a spectrophotometer (OD 340 nm) using SoftMax Pro 6.4 (Molecular Devices, San Jose, CA, USA) software.

### 2.5. Morphological Analysis of the Infection

SfMNPV OBs were obtained from the bioreactor samples centrifuged, as described above. Pelleted cells were dissolved with SDS 1% for 1 h at 28 °C. The OBs were counted in a Neubauer chamber under phase-contrast microscopy at a 400× magnification. Transmission Electron Microscopy of SfMNPV-infected cells was performed at 7 days post infection (dpi) with the bioreactor batch sample without culture medium supplementation to confirm the quality of the produced OBs. The samples were prepared according to [21]. The cell pellet was washed in phosphate-buffered saline (PBS) and immersed in a fixer (2.5% glutaraldehyde in 0.1 M sodium cacodylate, pH 7.3) at 4 °C overnight. Secondary fixation was carried out for 1 h with 1% osmium tetroxide. After fixation, the cells were dehydrated through an ascending acetone series, followed by a sequence of acetone: Spurr resin (3:1, 2:1, 1:1, and 5 mL of Spurr). Samples were kept in Spurr at 37 °C for 72 h. Ultrathin sections obtained with a Leica ultramicrotome using a diamond knife were stained with 2% uranyl acetate and visualized at the Zeiss TEM 109 electron microscope.

### 2.6. PCR Analysis

Bioreactor samples inoculated with MOI 0.1 (Run 3) were submitted to PCR analysis and sequencing to verify possible modifications in genes related to OB formation and structural nucleocapsid and envelope proteins. The OBs obtained from 0, 3, 5, and 7 dpi were dissolved in an alkaline solution and used for DNA extraction with DNeasy Blood & Tissue (Qiagen, Hilden, Germany), following the manufacturer’s instructions. The DNA was quantified with a low-DNA-mass ladder (Invitrogen, Waltham, MA, USA) in 0.8% agarose gel electrophoresis. SfMNPV-19 OBs obtained from larvae were extracted together. Specific primers were designed based on the SfMNPV-19 sequence [22] for *polh*, *protf*, *fp25k*, *pp34*, and *p10* genes (Table 1).

PCR was performed using GoTaq Flexi DNA Polymerase (Promega). The reaction for each gene was made with 1× PCR buffer, 0.4 mM dNTPs, 0.2 μM of primer pair, 2.0 mM MgCl_2_, 1.25 U Go Taq Flexi DNA polymerase, 1 μL DNA and RNAse-free water up to 50 μL. PCR was performed with an initial denaturation step at 95 °C, 5 min; then, 35 cycles of denaturation (95 °C, 30 s), annealing (50–64 °C, 1 min), and elongation (72 °C, 1 and a half minutes) and a final step of elongation occurred for 10 min. The PCR products were subjected to electrophoresis in 0.8% agarose gel [23]. The PCR products of 7 dpi samples were purified with a QIAquick gel extraction kit (Qiagen) according to the manufacturer’s instructions and sent for Sanger sequencing at Macrogen (Korea). The sequences were compared to the SfMNPV-19 sequence from GenBank (taxid accession number 10455) using ClustalW in the MEGA X program [24].

### 2.7. Quantitative Real-Time PCR (qPCR)

Samples from bioreactor inoculated with MOI 0.1 and MOI 1.0 were submitted to qPCR analysis to compare the viral DNA quantification to OBs quantification. The OBs obtained from 3 and 7 dpi (MOI 0.1) or at 3, 7, 11 and 14 dpi (MOI 1.0) were dissolved in an alkaline solution and used to extract DNA with DNeasy Blood & Tissue (Qiagen), following the manufacturer’s instructions. The DNA was quantified with a low-DNA-mass ladder (Invitrogen) in 0.8% agarose gel electrophoresis. SfMNPV-6nd OBs obtained from larvae were extracted and serially diluted for absolute quantification. The qPCR for SfMNPV with specific primers for the sf32 gene was carried out in a Rotor gene 5plex HRM platform (Qiagen), according to [17].

## 3. Results

### 3.1. Evaluation of Culture Media Supplementation for Sf9 Cells Growth

**Figure 1:**
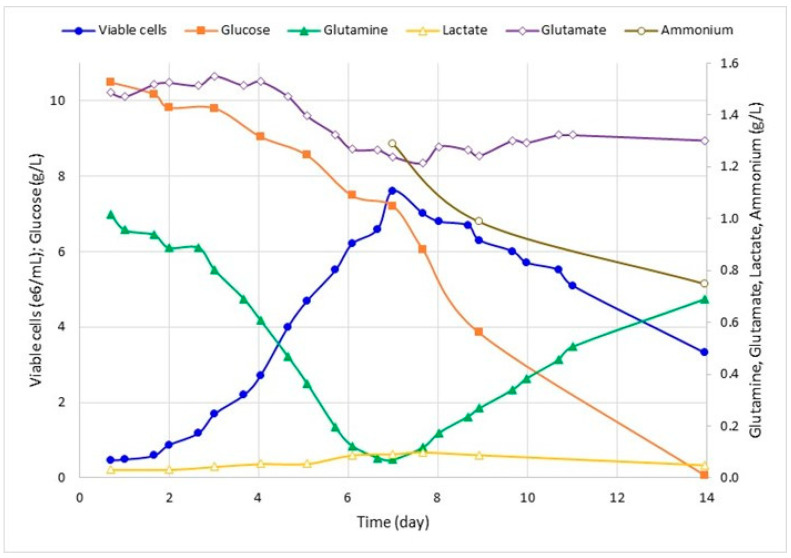
Kinetics of Run 1: Sf9 cell growth and metabolic analysis of glucose, lactate, glutamine, glutamate, and ammonium in STR bioreactor without glutamine supplementation. Samples were collected daily for cell-counting, expressed as viable cells ×10^6^/mL. Glucose, glutamine, and lactate were quantified and expressed as g/L.

**Figure 2:**
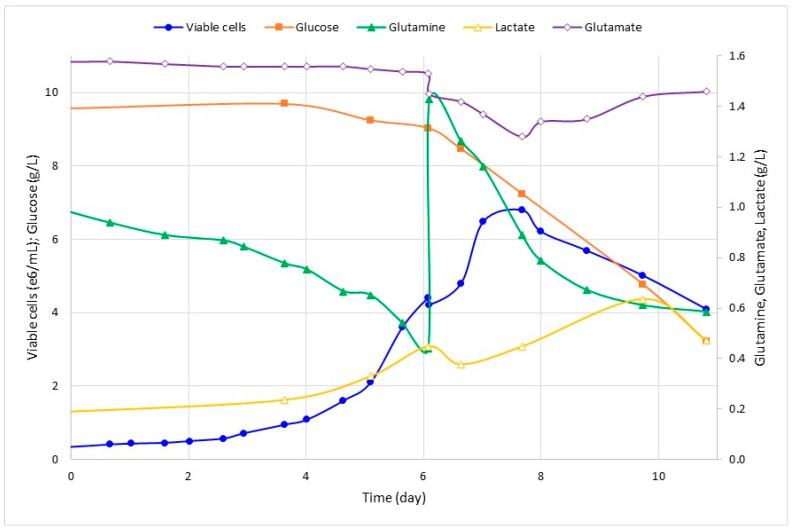
Kinetics of Run 2: Sf9 cell growth and metabolic analysis of glucose, lactate, glutamine, glutamate, and ammonium in STR bioreactor with glutamine supplementation at the end of the exponential growth phase. Samples were collected daily for cell-counting, expressed as viable cells ×10^6^/mL. Glucose, glutamine, and lactate were quantified and expressed as g/L. The time axis was shifted to adjust the growth lag phase between runs.

Run 1 was conducted to establish a metabolic reference for Sf-9 growth cultivation; hence, the metabolic analysis shows cell concentration peaks as glutamine is deployed (Figure 1). With this in mind, a new bioreactor run was designed to supplement glutamine as the growth reaches the exponential phase (Run 2, Figure 2). The comparison of metabolic analysis (Figure 1 and Figure 2) in the bioreactor batches with and without glutamine supplementation showed no clear effect of supplementation in the final values of viable cell concentration. The average final value of glutamine after supplementation was 0.48 g/L. This value is close to the glutamine concentration before supplementation. However, the substrate consumption rate was higher after supplementation, indicating nutritional necessity. Furthermore, without supplementation, after reaching glutamine depletion at the highest cell concentration, it seems to lead to a subsequent glutamine production and glucose consumption increase (Figure 1). With supplementation, this pattern is not observed, implying that the glutamine rate consumption may be more controlled and the growth may be less dependent on glucose (Figure 2). The other metabolic parameters (lactate, glutamate, and ammonium) presented a similar profile in both supplemented and non-supplemented culture media.

### 3.2. Effect of Viral Inoculum on SfMNPV Production

**Figure 3:**
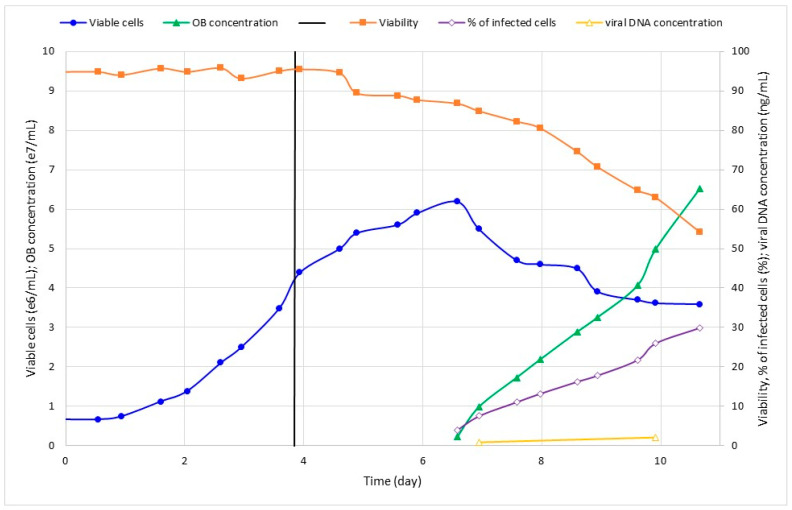
Kinetics of Run 3: Sf9 cell growing in glutamine–supplemented bioreactor and infected with SfMNPV-19 at day 3.9 (MOI 0.1). Samples were collected for cell-counting, expressed as viable cells/mL, and viability (%). OBs were counted and expressed as OBs/mL. Cells with OBs were expressed as % of infected cells. The time axis was shifted to adjust the lag growth phase between runs. The vertical line represents infection time.

**Figure 4:**
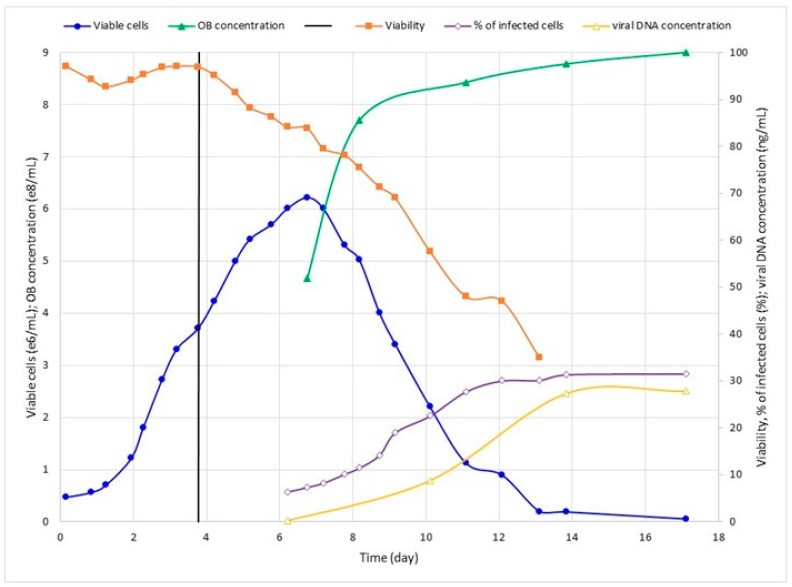
Kinetics of Run 4: Sf9 cell growth in glutamine-supplemented bioreactor and infected with SfMNPV-19 at day 3.8 (MOI 1.0). Samples were collected for cell-counting, expressed as viable cells/mL, and viability (%). OBs were counted and expressed as OBs/mL. Cells with OBs were expressed as % of infected cells. Viral DNA concentrations were expressed in ng/mL. The vertical line represents infection time.

In order to verify MOI’s influence on SfMNPV production, two different values (0.1 and 1.0) were tested in bioreactor with glutamine supplementation (Run 3 and Run 4, respectively). Cell concentration peaked after about 2.5 dpi as the cell viability dropped and infection progressed (Figure 3 and Figure 4). Run 4 was carried out for longer, but it is clear that, after about 10 dpi, cell infection was saturated even with the percentage of cell infection reaching around 30% (Figure 4). Additionally, this batch was used to test the viral DNA quantification through q-PCR, which showed a consistent correlation with other viral parameters (Figure 4).

In comparison, even though the maximum viable cell concentration and percentage of infected cells were similar in both experiments, the batch with a higher MOI presented with better viral yields (Table 2).

### 3.3. Quantitative PCR Standardization

**Figure 5:**
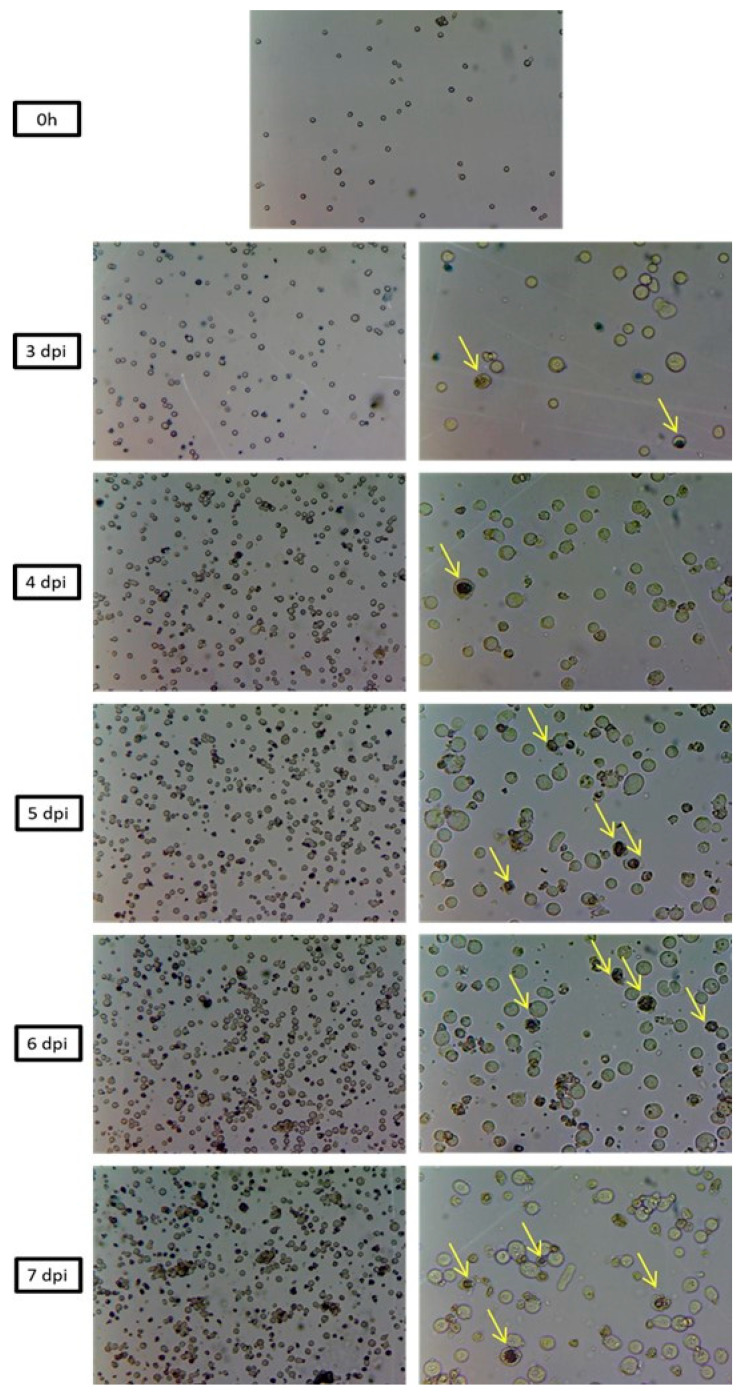
Optical micrographs with magnification of 64× and 160× of SfMNPV infection progress in Sf9 cells cultivated in STR bioreactor. 0 h (before SfMNPV infection). 3, 4, 5, 6, 7 days post-SfMNPV infection (dpi). The arrows point to the OBs inside the nuclei, which indicate morphological alterations induced by the virus.

To check the accuracy of qPCR quantification as a predictor for virus OBs production, the qPCR quantification of viral DNA was performed at 3, 7, 11 and 14 dpi in the bioreactor inoculated with MOI 1.0 (Run 4). The values were compared to optical microscopy OBs’ quantification at the same times post-infection (Figure 5). The curve in viral DNA presented a fast-growing rate from 3 to 11 dpi, and then stabilized it until 14 dpi. A similar pattern was observed for the curve of OBs/mL, as well as for the curve in the percentage of cells with OBs, indicating the accuracy of qPCR quantification and the best time for the viral production quantification.

### 3.4. Effect of SfMNPV Infection on Cell Metabolic Activities

The cell metabolic analysis of infected cells at 7 dpi (Table 3) revealed a higher glucose consumption by cells infected with MOI 1.0, evidencing an increase in cell consumption after the more efficient viral infection. Glutamine production was also higher in the cells inoculated with MOI 1.0 and, as a consequence, ammonium production was higher (Table 2 and Figure 4). Lactate metabolism was similar between MOIs, indicating that there was no shortness of oxygen [25]. On the other hand, the kinetics profile of glucose, lactate, glutamate, glutamine, and ammonium by non-infected and infected cells for 12 days was similar.

### 3.5. Sequencing Analysis of SfMNPV Produced in Bioreactor

The analysis of agarose gel electrophoresis of PCR fragments revealed that all the samples had the expected gene size (754 bp—*polh*, 1217 bp—*prot f*, 625 bp—*fp25k*, 1063 bp—*pp34*, 495 bp—*p10*), indicating that bioreactor production with MOI 0.1 did not cause selection for defective viruses in these essential structural genes. The nucleotide sequences of these samples did not present any mutation or indel that induced amino acid changes compared to the sequence of the wild SfMNPV-19 isolate.

### 3.6. Morphological Analysis of SfMNPV Production

**Figure 6:**
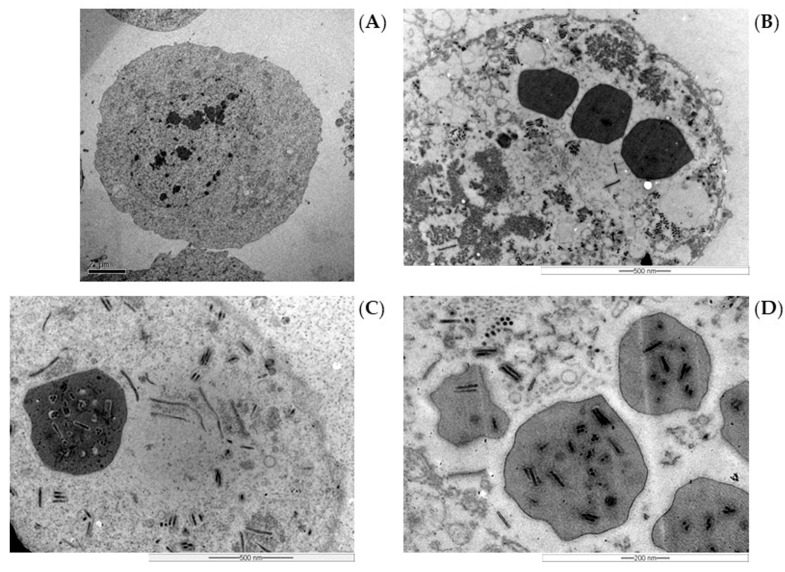
Electron micrographs of mock-infected Sf9 cells with 8000× magnification (**A**) and cells infected with SfMNPV-19 in STR bioreactor (**B**–**D**) at 7 dpi with 12,000×, 20,000× and 30,000× magnification, respectively. SEM performed with high voltage (HV) of 50,000 kV, horizontal field width (HFW) of 3.4 µm (**B**), 2.1 µm (**C**) and 1.4 µm (**D**).

The optical microscopy from 0 to 7 dpi in all bioreactor batches with SfMNPV revealed the progress of infection over time, with an increasing number of cells presenting typical baculoviruses infection symptoms, such as cell hypertrophy, cell lysis, and dark nuclei (Figure 5). Ultrastructural analysis of the infected Sf9 cells showed, as expected, cell nuclei hypertrophy and the production of many OBs compared to non-infected cells (control) (Figure 6). Other typical baculovirus-induced effects, such as the formation of virogenic stroma, nucleocapsids and virions, were also observed. No undesirable effects, such as few polyhedra in the cell nuclei or OB presenting aberrant polyhedra, were observed.

## 4. Discussion

At present, the production of SfMNPV for use as a commercial biopesticide is achieved by infecting healthy larvae [11]. This technique for mass production has disadvantages, such as the increased cost of the viral products due to the intensive labor required, cannibalistic larval behavior and contamination problems [12]. Baculovirus production in cell culture is an alternative to overcome such difficulties [13]. In vitro virus production in a bioreactor offers the possibility of achieving greater control of the process, with higher quality and greater volume in a smaller space/infrastructure.

There are several studies focused on the bioreactor culture of baculoviruses aiming to achieve recombinant protein production [26,27,28,29], although only a few studies have been conducted regarding the in vitro production of baculoviruses for pest control [30,31,32]. Advances were achieved in the selection of suitable cell lines for high OBs production [15,33]. However, there are still challenges to be overcome in the scale-up process for large-scale in vitro production. Several factors have been shown to affect the volumetric and specific polyhedra production in the Sf9 suspension culture infected with baculovirus. The initial cell seeding, MOI, and glucose consumption in infected cells are among the factors that were studied for AgMNPV infection in these cells [31]. For SfMNPV, studies of the kinetics of the infection in Sf9 growth were performed in suspension culture [14]. To our knowledge, the present study is the first to be carried out in a bioreactor for SfMNPV production.

We conducted preliminary studies with a combination of Sf9 cells, culture medium SF900 III and a SfMNPV isolate (named 19) that indicated the best initial cell seeding and the need for glutamine supplementation. The supplementation appeared to be necessary due to the coincidence between the end of the log phase of cell growth and the depletion of glutamine in the culture medium. The supplementation did not seem to impact the final cell density; however, it may play a more complex role in infected and non-infected cell metabolism. The accumulation of metabolic subproducts such as lactate and ammonium is closely related to oxygen and glutamine consumption [34]. Nevertheless, glutamine supplementation did not cause an excess of these toxic subproducts. The Sf9 cells’ efficiency in canalizing glucose to oxidation in the TCA cycle was noted, in addition to their ability to prevent the exaggerated formation of lactate [27,29]. On the other hand, the ammonium detoxication by the GS/GDH/GOGAT-recycling mechanism did not occur efficiently and caused the accumulation of glutamate, though nontoxic [35]. Finally, there are literature reports stating that the density effect directly affects viral production through a loss in metabolic efficiency dependent on the cell density of the culture and, consequently, greatly affects the stage of infection and viral production [36]. Low viral productivity at a high cell density has been observed, in conjunction with nutrient depletion and in controlled bioreactor cultures [25]. Thus, a better understanding of metabolic mechanisms can be very important to improve the rate of infection.

The ability to incorporate nutrients to sustain the viral division process was shown to be maintained without a reduction in consumption and general metabolism; that is, a specific metabolic adaptation occurs, and thus the specific consumption of glucose increases [37]. The intensification of post-infection metabolism is also supported by [38], who conducted a study of the metabolic flowchart and demonstrated that metabolism in Sf-9 cells is even more efficient via TCA after and during the viral infection process. In this study, an increase in the efficiency of glucose use for biosynthesis and post-infection energy production was observed.

The increase in the MOI for 1.0 (Run 4) resulted in a higher volumetric and specific production of SfMNPV, which resulted in a higher glucose consumption and, as a consequence, an increase in the lactate production by cells, but this did not reach critical values. The authors of [31] also observed an increase in glucose consumption and lactate production in Sf9 cells infected with AgMNPV, but did not observe any effects on glutamine consumption. They also observed a higher specific production with an increase in MOI of 0.1 to 1.0, although the volumetric production was lower.

The best approach observed in this work was achieved with the bioreactor using MOI 1.0, which resulted in a volumetric production of 9.0 × 10^8^ OBs/mL and a specific production of 340 OBs/cell.

Another concern regarding the in vitro production of baculoviruses is the virulence loss of the produced virions, since the presence of few polyhedra mutants, aberrant OBs morphology, few virions inside OBs and defective interfering genotypes has been reported [16,31,34,39]. The ultrastructural analysis and genetic analysis of several genes, performed for SfMNPV produced in the bioreactor, revealed OBs and virions without signals of critical modifications. However, the low virulence observed for OBs from static culture for this same isolate in another study [17] indicates the need for further work with bioassays and the complete genome sequencing of the isolates obtained from bioreactors.

Quantification by qPCR method, as performed in this work, was revealed to be a useful tool. It allowed for the prediction of OB values in suspension and, more importantly, the technique could help to compare different production batches with more accuracy, as well as preventing the intrinsic error caused by different people counting in the Neubauer chamber. Another advantage of the method is the possibility of quantification of the budded virus in the suspensions in conjunction with OBs, similar to the quantifications performed by [17].

## 5. Conclusions

Based on the presented results, we can conclude that the main factor of the infection process is to achieve a balance between an efficient MOI and the risk of generating mutants. Higher MOIs are more predictable, reproducible, and more adjustable through time of infection (TOI) [40], while lower MOIs tend to prevent mutant formation. The MOI of 1.0, despite presenting a similar percentage of cell infection as the MOI of 0.1, resulted in a specific productivity that was 10 times higher. This optimization enables high-scale production since it reached the minimum amount recommended by [13] for commercial production. A bioreactor with 300 L capacity, operating 20 times per year, with an average production of 10^12^ OBs/L, could produce enough virus to be applied to 60 thousand ha/year. Consequently, the in vitro-produced SfMNPV has the potential to protect the maize and other crops infested by fall armyworms. It is essential, however, that the produced OBs have a similar virulence to those obtained from larvae (in vivo). Further studies on the optimization process and evaluation of insect mortality (bioassays) by viruses produced in a bioreactor must be encouraged.

## Figures and Tables

**Table 1:** Sequences of specific primers designed *Spodoptera frugiperda multiple nucleopolyhedrovirus* (SfMNPV) genes, based on a complete sequence of SfMNPV-19 (GenBank accession code EU258200.1).

*Gene*	Primer Name	Primer Sequence (5′-3′)
*polh*	polhFw_Sf_2013 polhRv_Sf_2013	(AATGTATACTCGTTACAGCTATAACCCA) (GTGGTATGGTTTATTAGTACGCGGG)
*protf*	fproteinFw2_Sf_2013 fproteinRv2_Sf_2013	(GCCGAACGTAAGTTGTTGTT) (CATACACAGATCCATTAACATTTACA)
*fp25k*	25kfpFw_Sf_2013 25kfpRv_Sf_2013	(CATAAACTAACATGACGACTGCCACTG) (CGTTTATCGCGTTGCGCACTCATC)
*pp34*	pp34Fw_Sf_2013 pp34Rv_Sf_2013	(GTTACAATATAATGTCGTTGATTAC) (CTTGGATAATCCTTTGATTG)
*p10*	p10Fw_Sf_2013 p10Rv_Sf_2013	(CGCATTCGATTAGACGGACC) (GGCCACGATACAGAATTACGC)

**Table 2:** Maximal viable cell and SfMNPV (OB) concentrations in Sf9 cell cultures using two multiplicities of infection (MOI). Percentage of viable cells that were infected, relation between OB and infected cells and concentration of viral DNA at 7dpi in these runs.

Run	MOI	Maximum Viable Cell Concentration (×10^6^ cell/mL)	Maximum SfMNPV Concentrations (×10^6^ OBs/mL) (×10^7^)	Percentage of Infected Cells (%)	OBs/ Infected Cell	Viral DNA (ng/mL)
3	0.1	6.2	6.5	30.0	30.4	2.03
4	1.0	6.2	90.0	32.0	339	8.72

**Table 3:** Consumption or production rates of glucose, lactate, glutamate, glutamine, and ammonium by Sf9 cells infected with different multiplicities of infection (MOI) at 7 dpi.

Cell Metabolic Parameter	Cell Consumption/Production Rate (nmol/10^6^ cel/h)	
	MOI 0.1 (Run 3)	MOI 1.0 (Run 4)
Glucose	25.8	115.5
Lactate	0.9	1.7
Glutamine	9.8	12.3
Glutamate	2.7	1.5
Ammonium	0.9	13.4

## Data Availability

Not applicable.
